# Fear of falling: Scoping review and topic analysis using natural language processing

**DOI:** 10.1371/journal.pone.0293554

**Published:** 2023-10-31

**Authors:** Kamila Kolpashnikova, Laurence R. Harris, Shital Desai

**Affiliations:** 1 Social and Technological Systems Lab, York University, Toronto, Ontario, Canada; 2 Centre for Vision Research, York University, Toronto, Ontario, Canada; National Autonomous University of Mexico, MEXICO

## Abstract

Fear of falling (FoF) is a major concern among older adults and is associated with negative outcomes, such as decreased quality of life and increased risk of falls. Despite several systematic reviews conducted on various specific domains of FoF and its related interventions, the research area has only been minimally covered by scoping reviews, and a comprehensive scoping review mapping the range and scope of the research area is still lacking. This review aims to provide such a comprehensive investigation of the existing literature and identify main topics, gaps in the literature, and potential opportunities for bridging different strains of research. Using the PRISMA-ScR guidelines, we searched the Cochrane Database of Systematic Reviews, CINAHL, Embase, MEDLINE, PsycInfo, Scopus, and Web of Science databases. Following the screening process, 969 titles and abstracts were chosen for the review. Pre-processing steps included stop word removal, stemming, and term frequency-inverse document frequency vectorization. Using the Non-negative Matrix Factorization algorithm, we identified seven main topics and created a conceptual mapping of FoF research. The analysis also revealed that most studies focused on physical health-related factors, particularly balance and gait, with less attention paid to cognitive, psychological, social, and environmental factors. Moreover, more research could be done on demographic factors beyond gender and age with an interdisciplinary collaboration with social sciences. The review highlights the need for more nuanced and comprehensive understanding of FoF and calls for more research on less studied areas.

## Introduction

Fear of falling (FoF) is a major obstacle to active and independent aging among older adults [1–4]. FoF is defined as a persistent concern about falling down and the loss of self-efficacy [5] which is in turn connected with avoiding daily activities [6]. FoF prevalence varies between countries from 18% to 23% in the US [7, 8], 25% in Spain [4], and 17% in the EU countries [9]. According to the Center for Disease Control and Prevention, falls are one of the leading causes of injuries, including fatal injuries, in adults aged 65 years and older [10]. More than one in four older people have a fall in any given year [11]. In 2020 alone, preventable falls resulted in the deaths of 36,508 seniors aged 65 and above, with one-third of older adults having one or more falls inside their homes [12].

Among older adults who fall, an estimated 40–70% develop FoF as a consequence [6]. Additionally, many older adults who develop FoF have never fallen before [13–15]. Studies reveal that FoF considerably limits the quality of life among older adults [16–19] and has an adverse impact on balance confidence [20], social participation [21, 22], and engagement in physical activities [23, 24], among other health risks. The above makes FoF one of the major impediments to preserving autonomy, independence, and the ability to engage in everyday activities in older age.

### Rationale

Extensive general research on FoF has been done in previous research, particularly on its health-related determinants and outcomes [1, 2, 25, 26], such as balance [27–29], gait speed [30–32], depression [33, 34], comorbidities [35, 36], polypharmacy [37, 38], among many others. However, there has yet to be a comprehensive inquiry into mapping FoF studies and research interest areas, particularly with the inclusion of sociodemographic and environmental factors, with the exception of limited scoping reviews in health-related domains of FoF [39–43]. While some research has been conducted on separate domains of FoF, such as Parkinson’s disease [41], psychosocial responses [42], environmental factors [43], and common interventions [40], there has never been a comprehensive review to gauge the extent and range of research activity on FoF, including all of the above domains of FoF. This is partly due to the enormity of the task, with almost a thousand English-language research papers on FoF published today.

It is important to identify the range and scope of existing research on FoF for the following reasons. First, to this day, we lack a comprehensive interdisciplinary conceptual and empirical framework mapping associations (factors and outcomes) of FoF in a holistic manner. This scoping review develops a foundation for a comprehensive theoretical framework, providing a quick reference tool with such a conceptual mapping for scholars who are preparing literature or systematic reviews on FoF, As such, this review contributes to the theoretical investigations into concepts and factors of FoF, assisting scholars in their studies of FoF as a concept and enhancing our current theoretical and empirical understanding of FoF.

Second, the scoping review also enables identification of under-investigated factors related to FoF. Taking a bird’s eye view of FoF through a comprehensive scoping study helps us identify gaps in the current research. For example, while previous studies have established that visual information plays a crucial role in risk perception and FoF among older adults [44–46], only few intervention studies were actually carried out based on this knowledge. These studies include an intervention design using LED lights [47] and a research examining dim-light environmental conditions [48].

As the global population continues to age, the need to develop a comprehensive theoretical understanding of FoF, factors contributing to FoF, and its impact on the daily activities of older adults becomes increasingly important. Therefore, this scoping review is both timely and critical for research advancement in this area.

### Objective

The aim of this review is to map the existing research on FoF in older adults and identify main topics on FoF—all with the purpose of highlighting gaps and opportunities for future research and bridging existing areas of scientific inquiry. To achieve this, the review will address the following questions:

- What are the main topics that the FoF literature focuses on?
- What are the gaps in FoF research that need to be addressed?

## Methods

We conducted our scoping review using the Preferred Reporting Items for Systematic Reviews and Meta-Analyses extension for Scoping Reviews (PRISMA-ScR) guidelines [49] and a systematic subject heading and keyword search of scholarly databases. Specifically, we searched for the exact phrase ’fear of falling’ in articles’ titles and conducted subject heading and keyword searches on the population of interest (people aged 65 and over). This allowed us to narrow down our review to studies where FoF was the main focus.

The scoping review was registered with the Open Science Framework (https://osf.io/gyzjq). The protocol for the scoping review was published in BMJ Open [50].

### Eligibility criteria

We selected studies that analyzed FoF among older adults, both as a factor and an outcome variable. The sample included older adults living in their own homes, nursing homes, and assisted living arrangements. This review included automated topic analysis and researcher-assisted conceptual mapping, as outlined below. For the automated topic analysis, we included only original research, such as articles, dissertations, and conference papers, and excluded letters, editorials, or commentaries. The search included years from the inception date of each database to January 24th, 2023. Only works in English were considered. At the post-screening and the researcher-assisted analysis stage, we additionally excluded studies that did not analyze FoF as the focal concept. We identified only three such studies, which were all categorized together with poor quality abstracts. This study is a scoping review and as such does not require ethical approval.

### Information sources and search strategy

We included both quantitative and qualitative studies, but separated qualitative research in the researcher-assisted screening stage. No limitations were imposed on study design or date. Under the guidance of an experienced Health Sciences librarian, we conducted searches using both subject headings (e.g., MeSH) and keywords. We searched several databases, including the Cochrane Database of Systematic Reviews (CDSR), CINAHL, Embase, MEDLINE, PsycInfo, Scopus, and Web of Science. We first developed a MEDLINE search strategy and reviewed it with the three authors and the librarian. After creating the MEDLINE strategy, we adapted the subject headings and keyword searches to other databases. We used subject headings and keyword searches to define the population of the study. The search strategy for MEDLINE is presented in Table 1, and the search strategies for all other databases are included in the S1 Appendix. We used a mixture of subject headings (forward slash “/” at the end of a word or phrase in Table 1. represents MeSH) and keywords (ending with an .mp. suffix, which stands for “multi-purpose” to search for in title, original title, abstract, subject heading, name of substance, and registry word fields).

**Table 1:** MEDLINE (OVID) search.

MEDLINE search strategy
**1. "fear of falling".ti.**
**2. "aged, 80 and over"/**
**3. aged/**
**4. centenarians/**
**5. frail elderly/**
**6. nonagenarians/**
**7. octogenarians/**
**8. long-term care/**
**9. assisted living facilities/**
**10. homes for the aged/**
**11. nursing homes/**
**12. skilled nursing facilities/**
13. (Elder*).mp.
14. senior*.mp.
15. pensioner*.mp.
16. ((old* or aged*) adj3 (adult* or people* or person*)).mp.
**17. (Long-term care).mp.**
18. (nursing home*).mp.
**19. (assisted living).mp.**
**20. (home adj3 aged).mp.**
**21. or/2-20**
**22. 1 and 21**

### Study records and selection process

Given the vast number of included studies and the nature of our scoping review, aiming to identify overarching topics, we decided to automate the selection process with authors’ curation. The duplicates were detected through matching fields such as ‘author,’ ‘title,’ and ‘DOI’ in the search results. Additionally, we excluded entries that lacked titles or abstracts, that is, those that contained empty data in these fields.

To enhance our efforts to detect duplicate work, we utilized cosine and word2vec similarities in the titles of the studies. Word2Vec was particularly useful in identifying multiple reports of a single study. It is also worth noting that for our topic analysis, removing all duplicates at the screening stage was not necessary and would not bias the results considerably because of the large number of entries. The quality checks on each study for its inclusion or exclusion were not as critical prior to the topic analysis and were done at the post-screening stage, when authors analyzed all abstracts for subsequent conceptual mapping. This is because the aim of the scoping review is to identify the main overarching topics rather than to systematically review the results of the identified studies. The final search results were reported with a standard PRISMA flow diagram in the Results section.

In the post-screening stage following the topic analysis, authors identified studies of inadmissible quality (n = 12), as well as duplicates that were missed by automation (n = 2). This was done by researcher-assisted manual screening the abstracts within topics.

### Data collection process and data items

For this scoping review, we extracted titles and abstracts, which formed the primary data for the topic analysis. Automated extraction was used, as the selected studies’ tables already contained the required data (titles and abstracts), reducing the chances of errors during extraction. The full texts of the studies were not accessed.

The titles and abstracts of the included studies were combined into a single text for pre-processing. The pre-processing procedure involved several steps, such as restricting abstracts to conclusions and discussions, removing stopwords, stemming, and term frequency-inverse document frequency (TF-IDF) vectorization, using Python’s scikit-learn package. To restrict abstracts to discussions and conclusions, only the relevant sections of structured abstracts (n = 684) were extracted. This helped us focus on the main findings, rather than the entire abstracts. It is worth noting that this procedure was only applied to the portion of abstracts that had structured sections named Discussion(s) or Conclusion(s).

Stopword removal is the process of eliminating the words that do not contribute much to the meaning of the text from a text corpus to focus on the more informative words that convey meaning. During this stage, we removed a mixture of subject headings and keywords used in the database search, a curated stopword list [51], and uninformative words using an information-theoretic (conditional entropy) framework to eliminate additional words that were not informative in the analysis, using the method outlined in Gerlach and colleagues [52]. We also included diseases and conditions into stopwords, identifying them using a *scispacy* model (Python package). We removed medical conditions because they describe samples and populations rather than associations of FoF. However, in the synthesis of all associations of FoF, we added studies of different health conditions, which we identified in the researcher-assisted post-screening stage.

For stemming, we used Python’s *spacy* package for natural language processing (NLP) and only used nouns since we were interested in concepts and building a conceptual framework. Stemming is the process of reducing a word to its root form, commonly referred to as the “stem.” For example, the word “running” can be reduced to its stem “run.” Stemming allows for different variations of a word to be grouped together into a single word or a concept.

TF-IDF was used to transform the pre-processed texts into feature vectors, which were then used for topic modeling. It calculates statistical measures that reflect the importance of a word in a document corpus: 1) term frequency, which measures how often a word appears in a document, and 2) inverse document frequency, which measures how rare a word is in the corpus. The resulting score reflects the importance of a word in a particular document relative to the entire corpus.

### Data analysis

We used the Non-negative Matrix Factorization (NMF) algorithm for topic analysis [53], using Python’s scikit-learn package. NMF is an unsupervised machine learning algorithm commonly used for topic modeling in NLP. NMF decomposes a matrix X into two non-negative matrices (W, H), with the objective of minimizing the distance between X and the matrix product WH. For this paper, we used the Kullback-Leibler divergence, one of the most common objective functions for NMF [54], represented in the following formula:

$$D_{KL}(X|WH)=\sum_{i=1}^{m}\sum_{j=1}^{n}\left({\left(WH\right)}_{ij}-X_{ij}\log{\left(WH\right)}_{ij}\right)-\sum_{i=1}^{m}\sum_{j=1}^{n}\left(X_{ij}\;log\;X_{ij}-X_{ij}\right)$$

NMF has several advantages over other topic modeling algorithms, such as Latent Dirichlet Allocation (LDA). For example, NMF was found to be better-suited for mono-modal concepts (‘keywords only’) [55] and is faster than LDA [56].

## Results

### Search results

**Fig 1:**
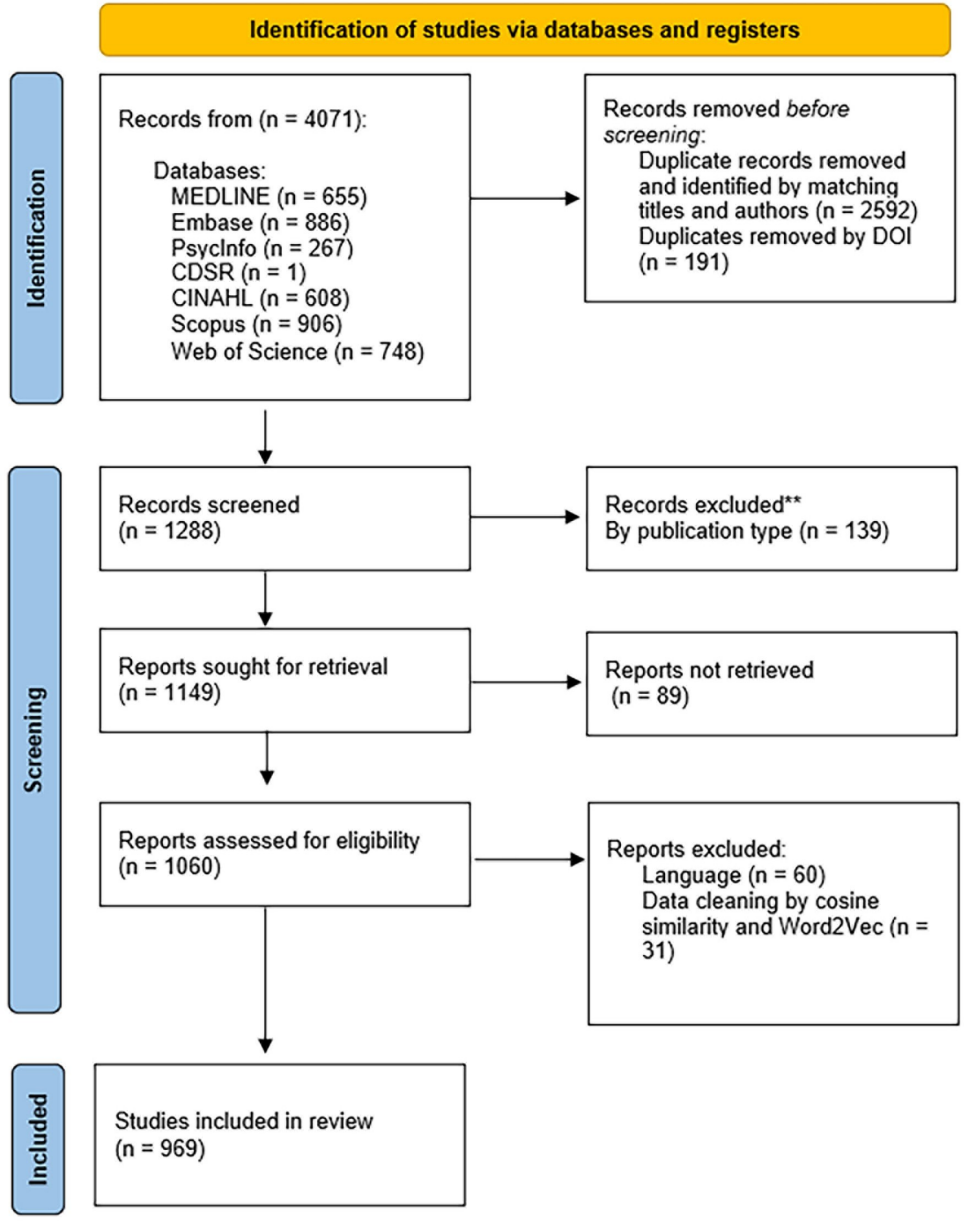
Schematic flow diagram for NLP analysis.

The initial database search yielded 4,071 entries. We eliminated duplicates by comparing titles and author names across different databases, resulting in a total of 2,592 entries. We also removed duplicates identified by DOI, amounting to 191 entries. During the screening process, we included only original research, such as articles, conference papers, book chapters, dissertations/theses, and proceedings papers, excluding 139 entries. We also removed entries without titles or abstracts (89 entries), as well as those not in English (n = 60). To further identify duplicates, we utilized cosine similarity and Word2Vec, resulting in an additional 31 entries being removed. Ultimately, we included 969 publications for the topic analysis. Fig 1 outlines the screening process and reasons for exclusion, following the Preferred Reporting Items for Systematic Reviews and Meta-Analyses (PRISMA) flow diagram.

### Topic analysis

**Fig 2:**
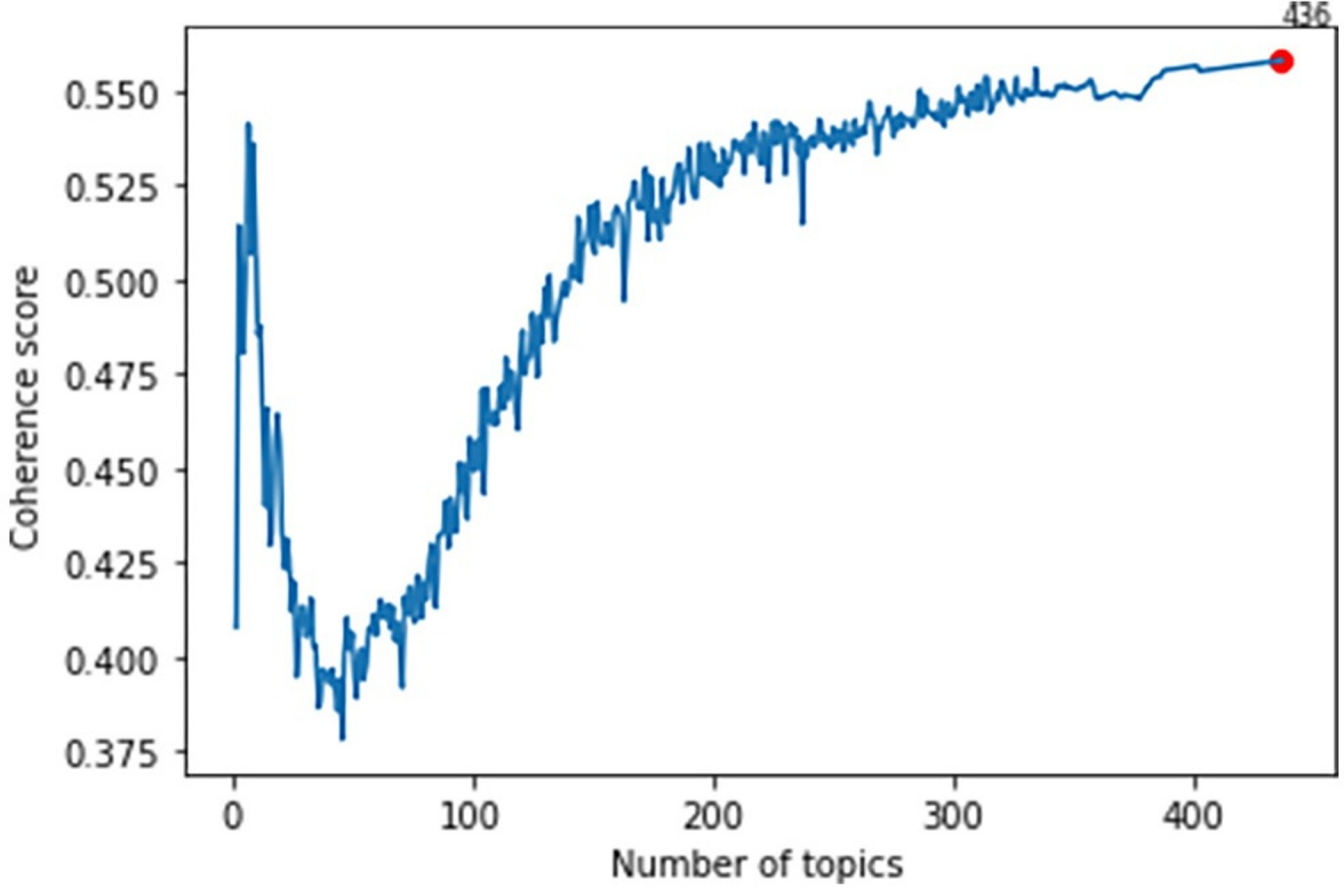
Topic coherence tests. The red dot represents the highest coherence score.

Using the NMF algorithm, we identified seven focal topics in the literature studying FoF. We selected the optimal number of topics based on the highest coherence score in the first modal half of the coherence score curve, as indicated in Fig 2. The coherence score was calculated using the *gensim* package.

**Fig 3:**
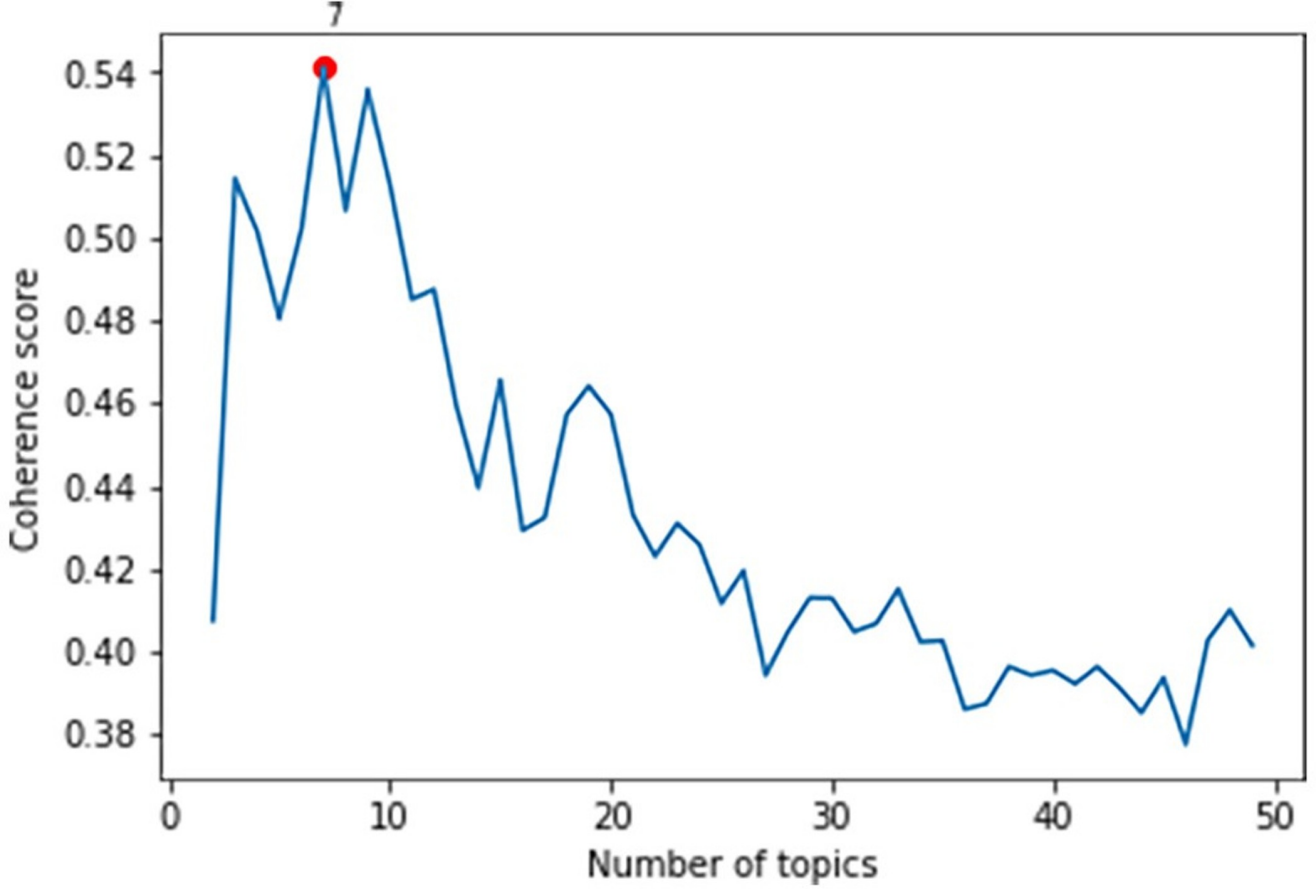
Topic coherence for <50 topics. The red dot represents the highest coherence score.

A conditional entropy-based stopword weeding algorithm, which was used in this study, results in a bimodal coherence curve that showed higher coherence scores for both a small and extremely large number of topics, up to the number of features minus one. The stopword removal algorithm was designed to reduce the number of topics within a large corpus, rather than relying on overfitting solutions found in the second half of the coherence scores curve in Fig 2 where coherence scores always improve with an increase in the number of topics. Thus, to choose the number of topics, we focused on the lower number of topics, corresponding to the left-side range in the coherence scores curve. Fig 3 shows the coherence scores graphed for the first modal part of the coherence score curve, and we observed the highest coherence scores at 7 topics. Therefore, we selected 7 topics and used the NMF algorithm to classify the texts of titles and abstracts accordingly.

**Fig 4:**
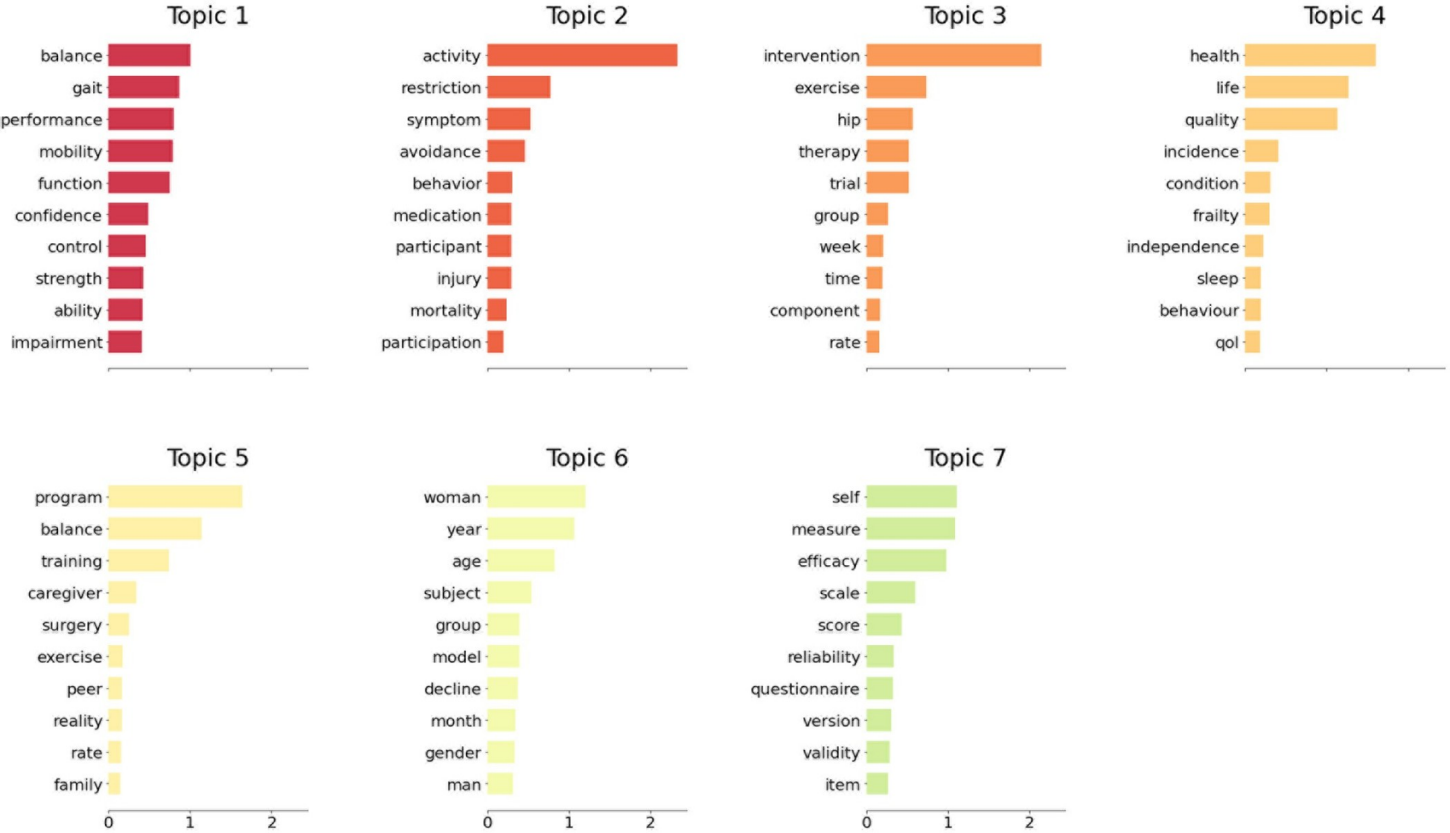
Major topics in FoF.

Fig 4 summarizes each of the 7 NMF-identified topics with the top 10 most common words. The largest core topics, Topics 1–3, contain studies on balance and gait, activity restriction, and intervention exercise studies. As Topic 5 also seemed to centralize training program interventions, it was ultimately combined with Topic 3 in the Topics Discussion section. Topic 4 represents concepts of higher levels of abstraction than in Topics 1 and 2, such as quality of life, health-related quality of life, and health care-related factors connected with FoF. Topic 6 brings demographic factors such as gender and age differences to the forefront, and Topic 7 centers around measurements and instruments of FoF. Although not all abstracts fit neatly into each topic, when grouped together, the topics formed provide us with information about the main foci of FoF research, such as balance and gait, activity restrictions, intervention studies, quality of life, gender and age, and FoF instruments.

**Fig 5:**
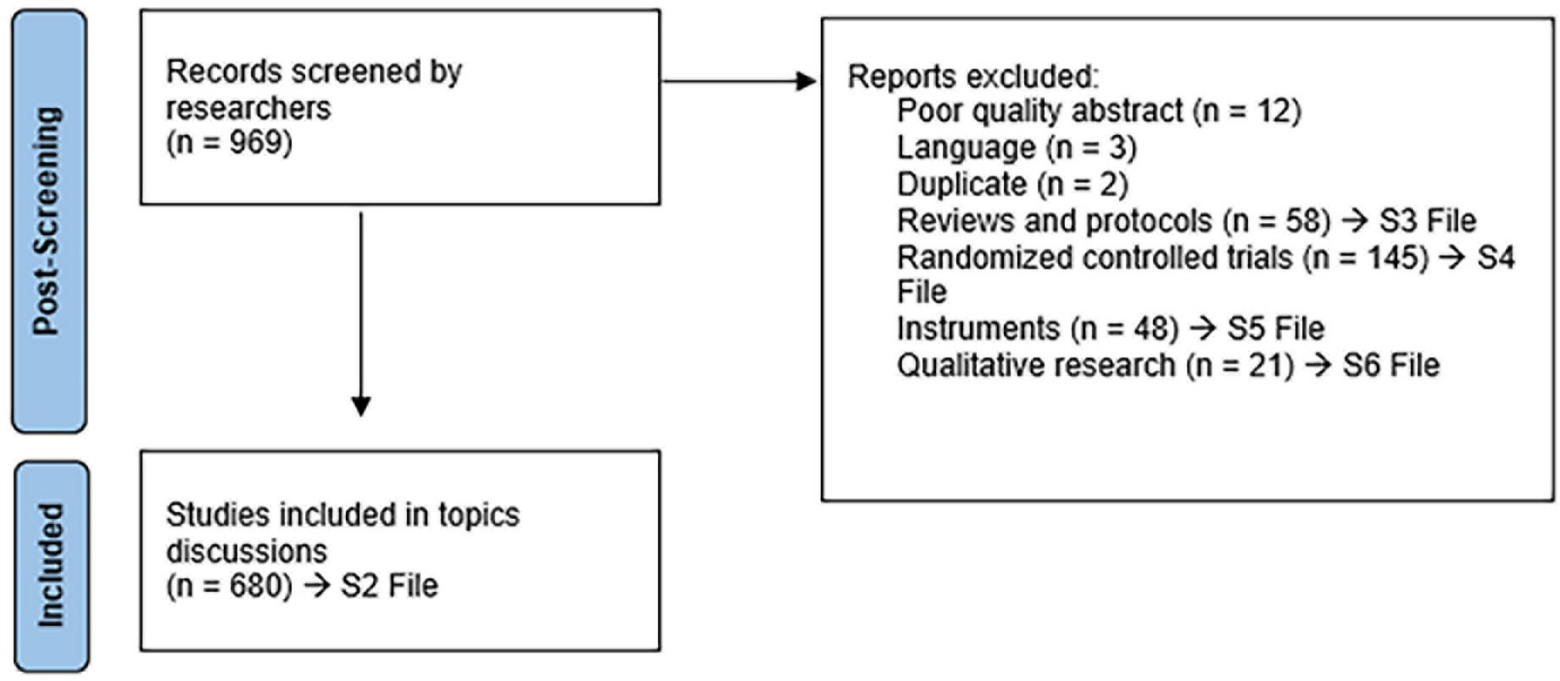
Flow diagram of post-screening by researchers.

After using machine learning automation to identify the focal topics, we conducted a researcher-assisted manual analysis of the abstracts in each group to determine the primary factor(s) associated with FoF in each paper. This resulted in a summarized synthesis table (S1 File) and the description of the topics and discussions that follow. Fig 5 summarizes the procedures of exclusion from the conceptual synthesis mapping. During this process, we also identified poor-quality abstracts (n = 12), abstracts of papers in another language (n = 3), and additional duplicated abstracts (n = 2). All included literature that contained different associations with FoF, i.e., the main data of this scoping review, is included in S2 File. The total of 680 studies were mapped in the conceptual framework in S1 File. Additionally, we created separate tables for systematic/scoping reviews and protocols (S3 File), randomized controlled trials (intervention studies and training programs) in Topics 3 and 5 (S4 File) and for Measurements and Instruments of FoF in Topic 7 (S5 File). Furthermore, we created a separate table for qualitative studies (S6 File). All these supplementary tables are created to assist scholars to easily access them for their research.

### Topics discussions

**Fig 6:**
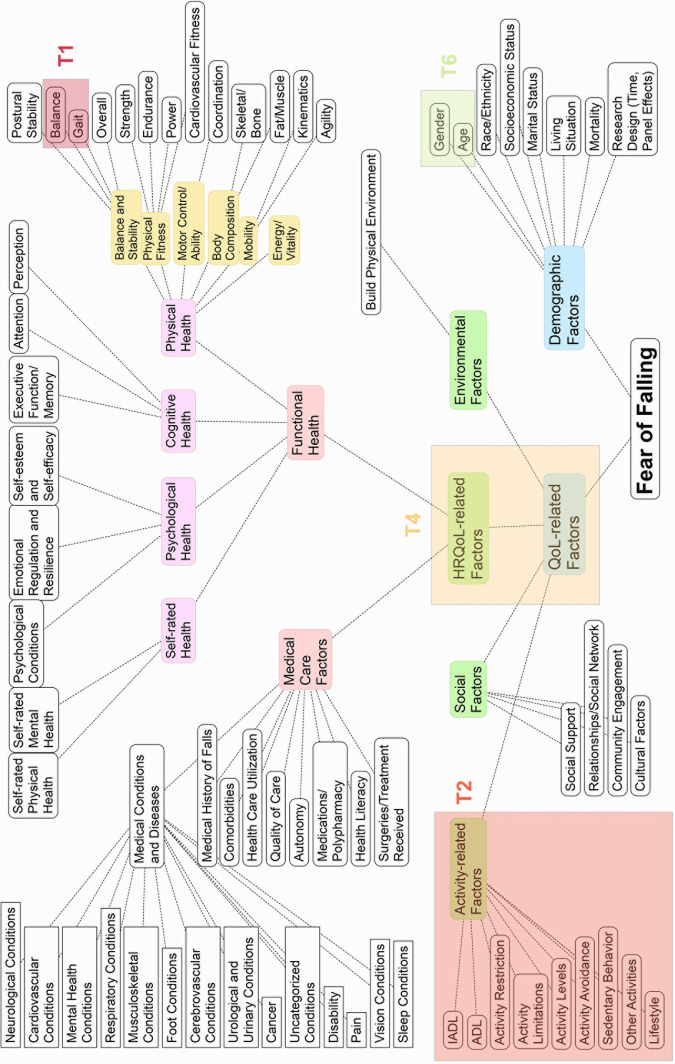
Summary of the conceptual mapping.

FoF factors comprised four focal topics. We discuss them in the following subsections. We present the ids of studies in parentheses and a prefix S2. For example, (S2: 351) can be found in S2 File with an id number 351. Full mapping of topics and concepts with corresponding study ids can be found in the conceptual mapping (see Fig 6; for details, see S1 File). Fig 6 summarizes the conceptual mapping and highlights the focal points within the FoF literature, as revealed in identified topics. It illustrates that certain broader concepts, such as quality of life and health-related quality of life, constitute their own topic (Topic 4, T4), encapsulating a majority of associated factors. In contrast, topics 1 and 6 indicate that specific factors, such as balance, gait, gender, and age, hold particular significance for FoF researchers.

Additionally, activity-related factors, which include activities of daily living (ADL) and instrumental activities of daily living (IADL), also form a central thematic area in FoF research. This topic also included biobehavioral conditions, such as activity avoidance, sedentary behavior, and other lifestyle-related factors.

Although medical conditions and diseases were included in the stopwords during the automated stage, we categorized works into broad categories of conditions in the second researcher-assisted stage of analysis (see Fig 6). The analysis revealed groupings of biomedical conditions, including neurological, cardiovascular, cancer, respiratory conditions, among others.

The conceptual mapping also reveals a relatively lower level of academic interest in social and environmental factors, as these do not emerge as prominent topics. Similarly, within the realm of functional health, there is a greater research focus on physical health factors and a comparatively less interest on cognitive and psychological health. Furthermore, medical care factors, such as health care utilization or quality of care, might warrant more attention in future FoF research.

### Topic 1: Balance and gait

This topic was the largest and contained a multitude of studies on physical health-related associations of FoF. There were many studies focusing on balance and almost equally as many on gait. These two factors of physical health, particularly those relating to balance and stability, were the focus of FoF research, suggesting that other factors related to physical fitness, motor control, body composition, mobility and vitality received less attention compared to these two.

Specifically, studies focused on different aspects and measures of balance, such as downward head pitches (S2: 351), balance control (S2: 352), dynamic balance control (S2: 352), balance impairment (S2: 602, 704), balance problems (S2: 278, 793), balance test (S2: 110, 514), instrumented Timed Up and Go (iTUG) scores (S2: 3, 545), TUG scores (S2: 235, 304, 514, 518, 545, 620, 874, 913, 928), one leg stand (S2: 442, 653), sideway velocity (S2: 389), stair ability (S2: 305), dynamic balance (S2: 235, 352) , balance loss recovery (S2: 519), and balance confidence (S2: 149). Similarly, in studying gait, the researchers looked at gait deficits (S2: 235), gait disorders (S2: 491), gait speed (S2: 145, 180, 245, 478, 502, 913, 930, 942), gait performance (S2: 46, 383), gait changes (S2: 218), gait variability (S2: 282), gait velocity (S2: 402, 506), freezing of gait (S2: 95, 506), cadence (S2: 502), slowness (S2: 38), step length (S2: 436), step time (S2: 502), stepping performance (S2: 507), number of steps (S2: 97), stride length (S2: 502), stride time variability (S2: 804), walking aid/cane (S2: 35, 52 , 112, 136, 156, 278, 429, 478, 504, 689, 693, 716, 743, 746, 816), walking capacity (S2: 294), walking endurance (S2: 597), walking speed (S2: 94, 517, 589, 653, 658), concentrate on each step while walking (S2: 482), foot and ankle characteristics (S2: 908), footwear (S2: 62), shoes (S2: 137), walking ability (S2: 302, 378, 648, 663, 947, 961), walk test (S2: 900), and walk time (S2: 877).

### Topic 2: Activity, activity restrictions, and activity avoidance

This topic included studies of ADL and IADL (S2: 214, 314, 379, 633, 638, 664, 871, 913). In line with health-related factors, this dimension of quality-of-life factors received more attention in FoF research when compared to social and environmental factors. These factors are related to the investigation of the consequences of FoF on ADL, IADL, and activity avoidance. Additionally, they encompass the self-perpetuating cycle of the association between diminished activity levels and FoF.

The topic also includes concepts of physical activity, activity restriction, activity limitation (S2: 45, 338, 429, 489, 490, 499, 572, 791, 819), activity levels (S2: 544, 647, 682, 722, 760, 968), and activity avoidance (S2: 233, 344, 400, 415, 418, 426, 434, 560, 672, 698, 790, 878). The topics also includes granular activities such as difficulty grocery shopping (S2: 195), difficulty in using public transport (S2: 278), exercise behavior (S2: 750), exercise (S2: 11, 367, 887), exercise expectations (S2: 901), exercise limitations (S2: 214), exercise needs (S2: 747), exercise regimen (S2: 747), being physically active (S2: 721), and time spent upright (S2: 238). It also includes lifestyle behaviors such as drinking status (S2: 939), driving habits (S2: 905), sedentary lifestyle (S2: 277, 516, 467), and sitting time (S2: 744).

### Topic 4: Quality of life, health-related quality of life, and health care factors

This topic approaches FoF from the most abstract level of factors (see Fig 6). Thus, it includes studies that focus on quality of life and health-related quality of life. But it also includes factors related to health and medical care, including medical history of falls and use of medications, etc. The observation that factors at a more abstract level constitute their own topic suggests that the research on FoF has not yet reached saturation in terms of the specificity of factors identified in relation to FoF within this domain, except for the balance and gait factors.

In the synthesis table (S1 File and Fig 6), this topic also includes all medical conditions and diseases identified in post-screening analysis. These conditions include neurological (Parkinson’s, dementia, palsy, polio, Ramsay Hunt syndrome, syncope, etc.), mental health (anxiety, depression, PTSD, OCD), cardiovascular (blood pressure, cardiac disease, hypertension), respiratory (COPD, dyspnea), musculoskeletal (osteoarthritis, rheumatoid arthritis, fractures, osteoporosis, sarcopenia, etc.), pain, cancer, among many others.

### Topic 6: Gender and age factors

Topic 6 is the only one that focuses on demographic factors, particularly on gender and age. The drawback of this topic is that many studies incorporate factors such as gender and age. However, this does not imply that they have theoretically developed explanations for the associations with FoF. While the connection between age and FoF seems self-evident, the theoretical link with gender is less clear.

Other less explored topics among other demographic factors were race and ethnicity (S2: 48, 278, 478, 548, 934), marital status (S2: 210, 541, 830, 939), household composition (S2: 214, 227, 333, 571, 572), and living in urban versus rural area (S2: 336). Some studies also looked at living alone (S2: 38, 115, 428, 444, 541, 638, 830, 924) and mortality (S2: 21, 32, 104, 113, 199, 771).

## Discussion

Our scoping review focused on the overarching topics emerging from the most researched factors associated with fear of falling (FoF). We found that most studies on FoF were concentrated on physical health-related factors, activity restrictions, and overall quality of life. Specifically, studies on balance and gait were more prevalent than studies on other physical health factors like mobility performance, flexibility, motor performance, body composition, or energy/vitality. Compared to the number of research on physical health, there were fewer studies on cognitive and psychological health.

Regarding quality of life, our study found that social and environmental dimensions of quality of life received less attention compared to the health-related dimension, which included physical, cognitive, and psychological health. Among social factors, studies paid more attention to social support than relational factors or community engagement. We also noted that there was a lack of research on cultural factors or social capital. This is an important finding because, in terms of structural and contextual information they provide, culture and social trust are critical in determining how people carry out their everyday activities. Among environmental factors, more attention was paid to factors of built environment, and none—to natural environments such as parks, quality of air, etc.

Furthermore, while gender and age were widely discussed, no other demographic factors were studied in-depth. Despite gender being identified as a focal factor in FoF, there were no systematic review specifically dedicated to analyzing gender differences among the 58 scoping and systematic reviews identified in our study.

In conclusion, our study provides a comprehensive overview of the current state of research on FoF. While physical health-related factors such as balance and gait remain the most extensively studied areas, our results suggest that other dimensions of physical health and social and environmental factors contributing to quality of life warrant greater attention. Similarly, our study highlights the need to explore the role of demographic factors such as race, ethnicity, and socioeconomic status in the study of FoF. By identifying gaps in the existing literature and highlighting under-researched areas, our study offers valuable insights for scholars and policymakers seeking to address FoF and its impact on individuals’ health and quality of life.

Compared to previous scoping reviews, especially those not specific to select medical conditions [39, 40], we have identified not only the overarching topics prevalent in the FoF literature, but also delved into sub-topics within them, organizing them into a structured taxonomy as presented in S1 Table in S1 Appendix and Fig 6. None of the previous scoping reviews of FoF have accomplished this. For example, in another comprehensive scoping review of FoF, MacKay and colleagues summarize all physical-health-related factors under a single thematic group “Physical Performance” [39]. In contrast, our review highlights that within physical-health-related factors, balance and gait take up the center stage. Moreover, we further break down physical-health-related factors into sub-topics such as physical fitness (individually for strength, endurance, power, and cardiovascular fitness), mobility and flexibility (individually for agility and kinematics), motor control and motor ability (including coordination), postural stability, body composition (separately for skeletal/bone composition and fat and muscle composition), as well as energy and vitality. This level of granularity surpasses that of any previous scoping reviews. This increased granularity enables the identification of gaps, even within the extensively researched area of physical-health-related factors. Previous studies, for instance, have not isolated factors like range of motion, flexibility, reaction time, and dexterity from overall physical performance. While range of motion is mentioned thematically in Chen and colleagues’ work [57], its focus is solely on its relationship to falls, not FoF. Additionally, whereas MacKay and colleagues identify 23 studies within this subtopic, our classification encompasses 462 studies within this overarching topic.

Based on our findings, there are several areas that could benefit from future research. Firstly, more research is needed on the cognitive and psychological aspects of FoF, as the extant literature focuses mostly on physical health-related factors. This could include investigating FoF cognitive and psychological factors apart from conditions such as depression and anxiety, which were studied extensively.

Secondly, there is a need for more research on the social and environmental factors that contribute to FoF. For example, examining the impact of community engagement, social trust, and cultural factors on FoF could provide a more comprehensive understanding of the phenomenon. By investigating these factors, researchers may be able to identify specific structural interventions or strategies that could be implemented to address the social and environmental factors that contribute to FoF, instead of focusing solely on individual-level factors like those of physical and cognitive performance.

Finally, there is also a need for more comprehensive research on demographic factors such as race, ethnicity, and socioeconomic status. By examining the potential disparities in FoF prevalence and outcomes among different demographic groups, researchers can gain a better understanding of the complex interplay between these factors and develop targeted interventions to reduce these disparities. For example, individuals from lower socioeconomic backgrounds may have limited access to healthcare and resources that could help them alleviate FoF, which could lead to poorer outcomes, overall. Similarly, older adults from certain racial or ethnic backgrounds may face unique cultural or linguistic barriers that impact their ability to seek help for FoF. By addressing these potential disparities, we can work towards a more equitable and inclusive approach to addressing FoF and improving the overall health and well-being of older adults.

There are several limitations to this study. First, the search was restricted to English language publications, which may have resulted in relevant literature being excluded. Particularly, there are a lot of studies on FoF done in Japanese. In the future, we plan to conduct a similar scoping review with the inclusion of works in Japanese with the help of our collaborators.

Second, only literature containing specific keywords in the abstract were included in the manual analysis and synthesis for identifying associations of FoF. This could have led to the exclusion of relevant literature that did not include those keywords in the abstract, particularly if the abstracts were not well-structured and did not accurately represent the key findings of the study. Future studies could consider broader inclusion criteria and more thorough screening methods to minimize the risk of missing relevant literature. Additionally, conducting a full-text review of potentially relevant articles could also help to ensure that important information is not overlooked. However, such an undertaking would be very difficult given the volume of existing literature on FoF.

Finally, due to the fact that the aim of the scoping review was to provide a fuller scope and range of research on FoF, the study did not assess the quality of the included literature, which may have given the same valence to quality research as well as to less-rigorous studies. In future research, it would be beneficial to conduct a more rigorous quality assessment, in order to ensure that the conclusions drawn are based on high-quality evidence.

Overall, our study highlights the need for a more comprehensive understanding of FoF and associated factors. We urge researchers to explore less studied areas and investigate cognitive, psychological, social, and environmental aspects, as well as cultural and demographic factors, to develop more effective interventions and improve the quality of life for older adults.

## Supporting information

S1 File Synthesis and conceptual mapping of the research on FoF. (XLSX)

S2 File Main data and articles’ ids included in the scoping review and conceptual mapping. (XLSX)

S3 File List of systematic and scoping reviews and protocols. (XLSX)

S4 File List of randomized controlled trials (intervention studies and training programs). (XLSX)

S5 File List of studies on measurements and instruments of FoF. (XLSX)

S6 File List of qualitative studies. (XLSX)

S1 Checklist Preferred Reporting Items for Systematic reviews and Meta-Analyses extension for Scoping Reviews (PRISMA-ScR) checklist. (PDF)

S1 Appendix Search strategies. (DOCX)
