# Supplementary material for: Fear of falling: Scoping review and topic analysis using natural language processing
Source: PLoS One. 2023 Oct 31;18(10):e0293554. doi: 10.1371/journal.pone.0293554 (PMC10617702; doi:10.1371/journal.pone.0293554)
Supplement: S1 Appendix — (DOCX) [file pone.0293554.s008.docx]

# APPENDIX

## Search Strategies

Database: Medline (1946 to 2023)

| Table S1. MEDLINE (OVID) search |
| --- |
| 1. "fear of falling".ti. 2. "aged, 80 and over"/ 3. aged/ 4. centenarians/ 5. frail elderly/ 6. nonagenarians/ 7. octogenarians/ 8. long-term care/ 9. assisted living facilities/ 10. homes for the aged/ 11. nursing homes/ 12. skilled nursing facilities/ 13. (Elder*).mp. 14. senior*.mp. 15. pensioner*.mp. 16. ((old* or aged*) adj3 (adult* or people* or person*)).mp. 17. (Long-term care).mp. 18. (nursing home*).mp. 19. (assisted living).mp. 20. (home adj3 aged).mp. 21. or/2-20 22. 1 and 21 |

Database: Embase (1974 to 2023)

| Table S2. Embase (OVID) search |
| --- |
| 1. "fear of falling".ti. 2. "aged, 80 and over"/ 3. aged/ 4. aged hospital patient/ 5. frail elderly/ 6. institutionalized elderly/ 7. very elderly/ 8. long term care/ 9. assisted living facility/ 10. home for the aged/ 11. elderly care/ 12. nursing home/ 13. nursing home patient/ 14. (Elder* or senior* or pensioner* or ((old* or aged*) adj3 (adult* or people* or person*))).mp. 15. (Long-term care or nursing home* or assisted living or (home adj3 aged)).mp. 16. or/2-15 17. 1 and 16 |

Database: PsycInfo (1806 to 2023)

| Table S3. PsycInfo search |
| --- |
| 1. "fear of falling".ti. 2. aging/ 3. long term care/ 4. adult day care/ 5. nursing home residents/ 6. exp nursing homes/ 7. retirement communities/ 8. assisted living/ 9. (Elder* or senior* or pensioner* or ((old* or aged*) adj3 (adult* or people* or person*))).mp. 10. (Long-term care or nursing home* or assisted living or (home adj3 aged)).mp. 11. or/2-10 12. 1 and 11 |

Database: EBM Reviews - CDSM (2005 to 2023)

| Table S4. CDSM search |
| --- |
| 1. "fear of falling".ti. 2. (Elder* or senior* or pensioner* or ((old* or aged*) adj3 (adult* or people* or person*))).mp. 3. (Long-term care or nursing home* or assisted living or (home adj3 aged)).mp. 4. or/2-3 5. 1 and 4 |

Database: CINAHL

| Table S5. CINAHL search |
| --- |
| 1. TI "fear of falling" 2. (MH "Aged") OR (MH "Aged, 80 and Over") OR (MH "Aged, Hospitalized") OR (MH "Frail Elderly") OR (MH "Centenarians") 3. (MH "Long Term Care") OR (MH "Assisted Living") OR (MH "Housing for the Elderly") OR (MH "Skilled Nursing Facilities") OR (MH "Nursing Homes") OR (MH "Nursing Home Patients") 4. (Elder* or senior* or pensioner* or ((old* or aged*) N3 (adult* or people* or person*))) 5. (Long-term care or nursing home* or assisted living or (home N3 aged)) 6. 2 OR 3 OR 4 OR 5 7. 1 AND 6 |

Database: Scopus

| Table S6. Scopus search |
| --- |
| 1. TITLE-ABS-KEY ( {fear of falling} ) AND ALL ( ( elder* OR senior*or AND pensioner* OR ( ( old* OR aged* ) W/3 ( adult* OR people* OR person* ) ) ) OR ( ( long-term AND care ) OR ( nursing AND home* ) OR ( assisted AND living ) OR ( home W/3 aged ) ) ) |

Database: Web of Science

| Table S7. WoS search |
| --- |
| 1. TI="fear of falling" 2. ALL=(Elder* or senior* or pensioner* or old* adult* or aged* adult* or old* people* or aged* people* or old* person* or aged* person* or Long-term care or nursing home* or assisted living or home for aged) 3. 1 AND 2 |
